# Structure, Magnetocaloric Effect and Critical Behavior of the Fe_60_Co_12_Gd_4_Mo_3_B_21_ Amorphous Ribbons

**DOI:** 10.3390/ma15010034

**Published:** 2021-12-21

**Authors:** Agnieszka Łukiewska, Piotr Gębara

**Affiliations:** Department of Physics, Częstochowa University of Technology, Armii Krajowej 19 Av., 42-201 Czestochowa, Poland

**Keywords:** amorphous alloys, microstructure, magnetic properties, Curie temperature, magnetocaloric effect

## Abstract

The aim of the paper was to study the structure, magnetic properties and critical behavior of the Fe_60_Co_12_Gd_4_Mo_3_B_21_ alloy. The X-ray diffractometry and the Mössbauer spectroscopy studies confirmed amorphous structure. The analysis of temperature evolution of the exponent n (ΔS_M_ = C·(B_max_)^n^) and the Arrott plots showed the second order phase transition in investigated material. The analysis of critical behavior was carried out in order to reveal the critical exponents and precise T_C_ value. The ascertained critical exponents were used to determine the theoretical value of the exponent n, which corresponded well with experimental results.

## 1. Introduction

The Fe-Co-B-based amorphous alloys are interesting from a basic research point of view because they do not show any topological order in the structural arrangement of the component atoms. These alloys exhibit high magnetic saturation, high magnetic permeability and extremely low coercivity, and these properties classify them as soft magnetic materials [1,2]. It is well known that their structure and properties can be changed by additions of such atoms as Mo, Gd, [3], etc. or structural modifications that are introduced during their fabrication. Substitution of Fe atoms of non-magnetic atoms such as Mo has been shown to bring changes in the magnetic properties of alloys [4]. These substitutions for Fe are reported to bring in T_C_ enhancement, which is largely associated to the magnetovolume effect altering the strengthening of the Fe–Fe exchange interactions [5]. Moreover, the Mo content is the important factor, which influences the thermal stability and saturation induction value [6]. Moreover, the atomic radius of the Mo is larger than Fe, which improves the glass forming ability. The pure Gd is natural magnetocaloric material with relatively high magnetic moment of about 7 μ_B_. Many magnetocaloric materials are based on the Gd, which were widely presented in [7,8]. As it was shown by Law and co-workers in [9], the successive addition of Gd to Fe-based alloy increased the Curie temperature and caused decrease of magnetic entropy change. The Fe-based alloys are a good candidate to be an active magnetic regenerator in magnetic refrigerators or heat pumps. In cases of amorphous Fe-based alloys, they are especially good for this application due to an occurrence of second order phase transition, which is related to relatively easy remagnetization and lack of temperature hysteresis. In this paper, we presented the magnetic properties, crystallization process and structure of the Fe_60_Co_12_Gd_4_Mo_3_B_21_ alloy.

## 2. Materials and Methods

The ingot sample was prepared by arc-melting of an appropriate amount of high purity elements (3N and higher) under the protective argon atmosphere of Ar gas. For ensuring their homogeneity, the samples were remelted several times. Amorphous ribbon of Fe_60_Co_12_Gd_4_Mo_3_B_21_ alloy, 2 mm wide and 25 μm thick, was fabricated by the melt-spinning technique using precursor ingots. The structure of produced specimens was checked by a Bruker D8 Advance X-ray diffractometer with CuKα radiation (Bruker, Karlruhe, Germany). The thermal stability of the amorphous alloys was investigated by differential scanning calorimetry (DSC) using NETSCH STA 449F1 Jupiter (NETZSCH Analysing & Testing, Ahlden, Germany) set at the heating rate of 10 K/min. The structure and magnetic characteristic of the samples were studied by Mössbauer spectroscopy using a Polon spectrometer. The transmission Mössbauer spectra were recorded at room temperature by means of a conventional constant acceleration spectrometer (POLON, Bydgoszcz, Poland) with a ^57^Co(Rh) radioactive source. The spectrometer was calibrated and the isomer shift (IS) was determined with respect to the polycrystalline α-Fe foil. Spectra fittings were carried out using the Normos package (written by R. A. Brand) according to the procedure developed in [10]. The thermomagnetic curves were recorded using vibration sample magnetometer (VSM) VersaLab Quantum Design system (Quantum Design, San Diego, CA, USA) in the temperature range from 50 K up to 400 K at the magnetizing field induction of 5 mT. The magnetocaloric effect was studied indirectly based on M vs. H curves collected in wide range of temperatures.

## 3. Results

**Figure 1:**
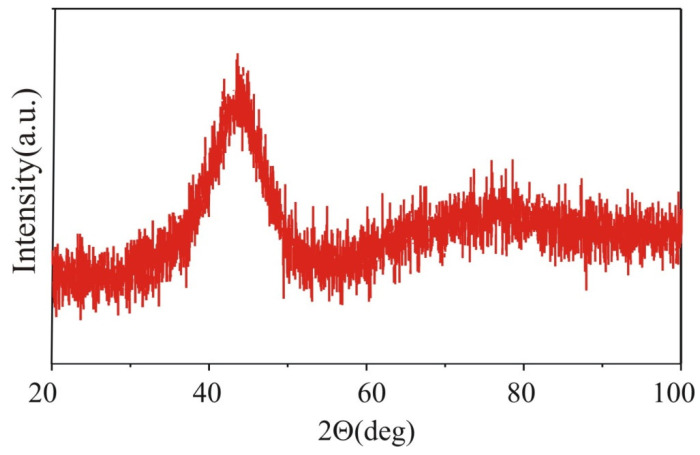
The X-ray pattern of the as-cast Fe_60_Co_12_Gd_4_Mo_3_B_21_ alloy ribbon.

**Figure 2:**
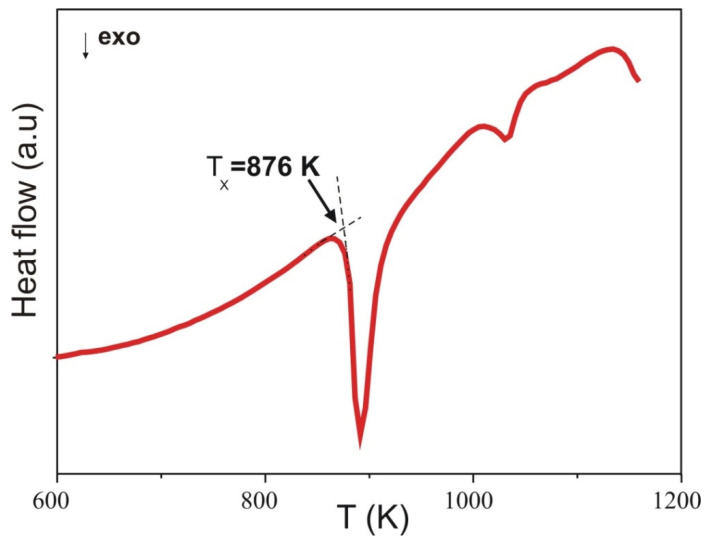
DSC curves for investigated Fe_60_Co_12_Gd_4_Mo_3_B_21_ alloys. Tx: the onset of the primary crystallization.

**Figure 3:**
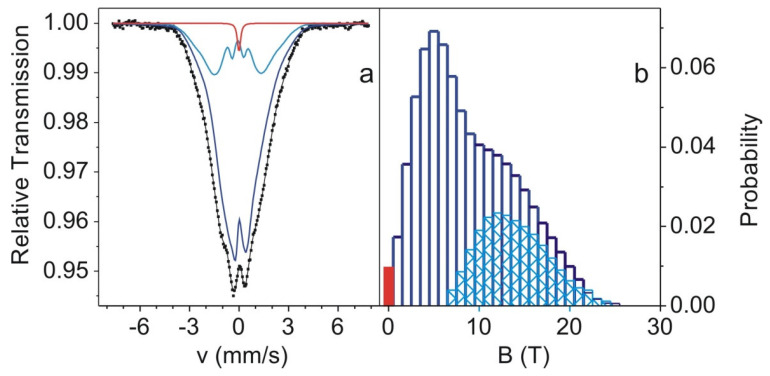
Transmission Mössbauer spectra (**a**) and corresponding hyperfine field distributions (**b**) for the Fe_60_Co_12_Gd_4_Mo_3_B_21_ alloy.

In Figure 1, the X-ray pattern is shown. The characteristic amorphous halo is clearly observed at 35–50°. The position of maximum of broad hump is in the vicinity of 44°, which is typical for the Fe-based alloys. An existence of broad hump and lack of the Bragg reflexes confirmed the amorphous structure of the prepared samples. The results of DSC measurements for the Fe_60_Co_12_Gd_4_Mo_3_B_21_ alloy are shown in Figure 2. The investigated alloy exhibits several exothermic peaks, indicating that the crystallization process is multistage. The first peak corresponds to the primary crystallization. The second and next peaks are related to the crystallization of the remaining amorphous matrix. In Figure 3, the transmission Mössbauer spectrum and corresponding distributions of hyperfine field induction of the as-cast Fe_60_Co_12_Gd_4_Mo_3_B_21_ alloy ribbon are depicted. The presented spectrum has the form of asymmetric lines and consists of an elementary spectra derived from ^57^Fe atoms with different surroundings. For this reason the spectrum was decomposed into two subspectra, which were the sum of elementary sextets with the hyperfine field induction (B_hf_)_1_, (B_hf_)_2_ and isomer shift (IS_0_)_1_, (IS_0_)_2_ described by the linear relation:

(B_hf_)_i_ = (B_hf_)_0_ + iΔB and (IS_0_)_i_ = (IS_0_) + aB_i_ (1)

where ΔB = 1 T is the step of the hyperfine field induction change and a is the directional coefficient of the straight line.

**Figure 4:**
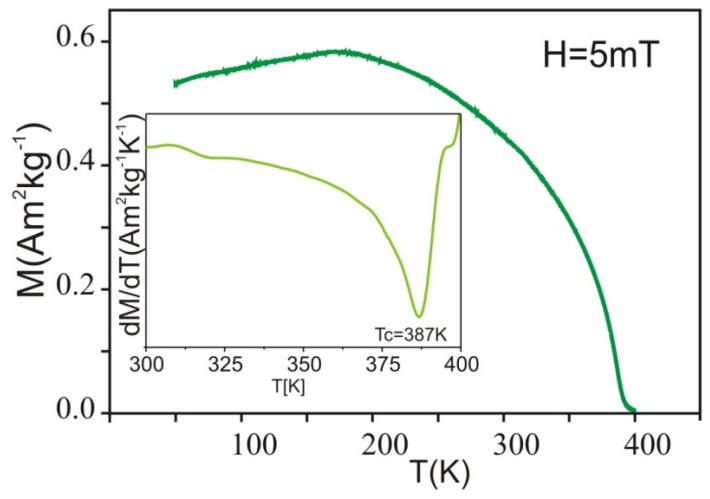
Specific magnetization, M measured at μ_0_H = 5 mT, as a function of temperature for the Fe_60_Co_12_Gd_4_Mo_3_B_21_ alloy and the derivative dM/dT versus temperature (in insert).

All parameters of the Mössbauer spectrum fitting are listed in Table 1. The hyperfine field induction distribution for the first subspectrum (not hatched area in Figure 3b) corresponds to areas of the high concentration of non-magnetic atoms. The second subspectrum (hatched area) in the hyperfine field induction distribution is related to the increased concentration of Co, Fe, Gd atoms in the nearest ^57^Fe atoms neighborhood. For the Fe_60_Co_12_Gd_4_Mo_3_B_21_ alloy, one can observe very small areas (about 1% of area of the spectrum) with B_hf_ = 0. The Curie temperature for investigated alloy was obtained with numerically calculated derivatives from M(T) curves. The result of calculation for the Fe_60_Co_12_Gd_4_Mo_3_B_21_ is shown in Figure 4. The Curie temperature for the Fe_60_Co_12_Gd_4_Mo_3_B_21_ alloy is T_C_ = 387 K (+2 K).

In order to determine the magnetic entropy change, the thermomagnetic curves M vs. H were collected for a wide range of temperatures. Taking into account the thermomagnetic Maxwell relation [11]:

(2) $$\Delta S_{M}(T,\Delta H)=\mu_{0}\int_{0}^{H}{\left(\frac{\partial M(T,H)}{\partial T}\right)}_{H}dH,$$

where T, μ_0_, H and M are mean temperature, magnetic permeability, magnetic field and magnetization, respectively.

Taking into account that Equation (2) could be rewritten in as the following Algorithm (3), which was implemented in Mathematica package [12]:

(3) $$\Delta S_{M}(\frac{T_{i}+T_{i+1}}{2})\approx \frac{1}{T_{i+1}-T_{i}}\left[\int_{0}^{B_{\max}}M(T_{i+1},B)dB-\int_{0}^{B_{\max}}M(T_{i},B)dB\right]$$

where B is magnetic field induction, according to relation B = μ_0_H.

**Figure 5:**
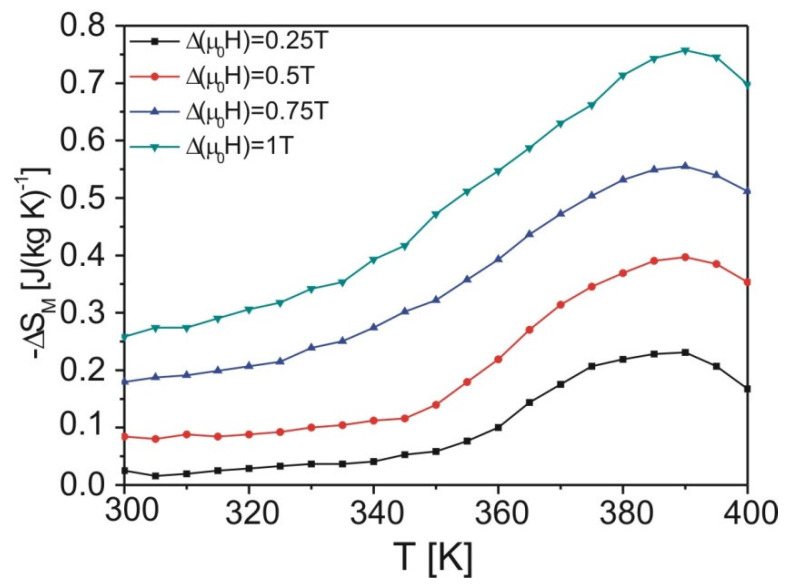
The temperature dependence of magnetic entropy change of the as-cast Fe_60_Co_12_Gd_4_Mo_3_B_21_ alloy.

Figure 5 depicts temperature dependence of magnetic entropy change. Broad maximum is typical for amorphous materials. The maximum value of the ΔS_M_ equaled 0.76 J (kg K)^−1^ under the change of external magnetic field Δ(μ_0_H) = 1 T. This values is higher or comparable than those presented in [9,13,14]

**Figure 6:**
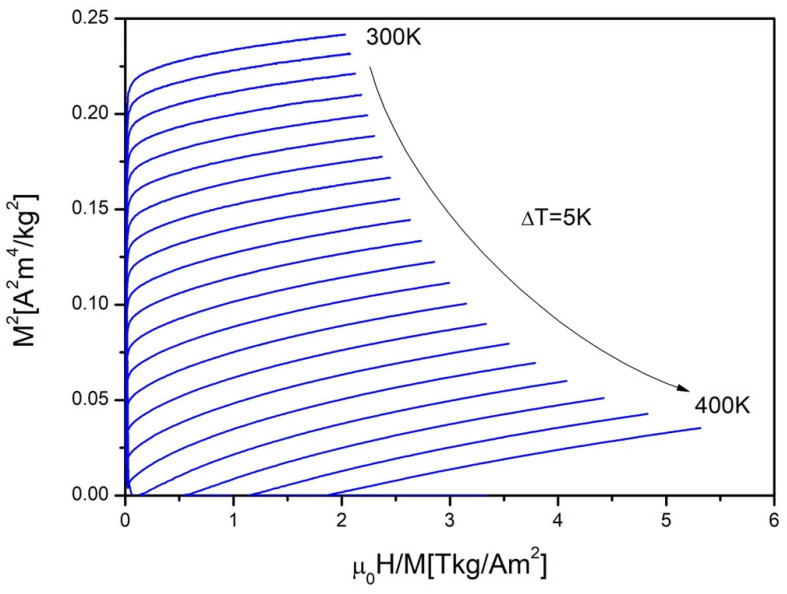
The Arrott plots constructed for studied material.

Figure 6 plots thermomagnetic isotherms M^2^ vs. (H/M) called Arrott plots obtained near the Curie temperature of the studied sample. The Arrott plots have positive show and show almost straight lines. The Banerjee criterion [15] describes differences in slope of the Arrott plots and its relation with order of the phase transition. The shape of Arrott plots, presented in Figure 6, confirms second order phase transition of the investigated alloy.

As it is well known that the magnetic entropy change increases with an increase of an external magnetic field (see Figure 5), Franco and co-workers in [16] proposed the phenomenological formula describing field dependence of the magnetic entropy change, which is given as:

(4) $$\Delta S_{M}=C\cdot {\left(B_{MAX}\right)}^{n}$$

where C is a constant depending on temperature and n is the exponent related to the magnetic state of specimen.

Świerczek in [17] showed how easy it was to reveal the n exponent by rewriting Equation (4) in following form:

(5) $$\ln\Delta S_{M}=\ln C+n\ln B_{MAX}$$

Franco et al. in [16] described that the n exponent is strongly dependent on the magnetic state. Taking into account that material obeys the Curie law, the exponent n equals 1 in ferromagnetic state (<T_C_), while n = 2 in paramagnetic state (>T_C_). Moreover, the exponent n value at the Curie temperature is given by the relation:

(6) $$n=1+\frac{1}{\delta \left(1-\frac{1}{\beta }\right)}$$

where β and δ are critical exponents.

**Figure 7:**
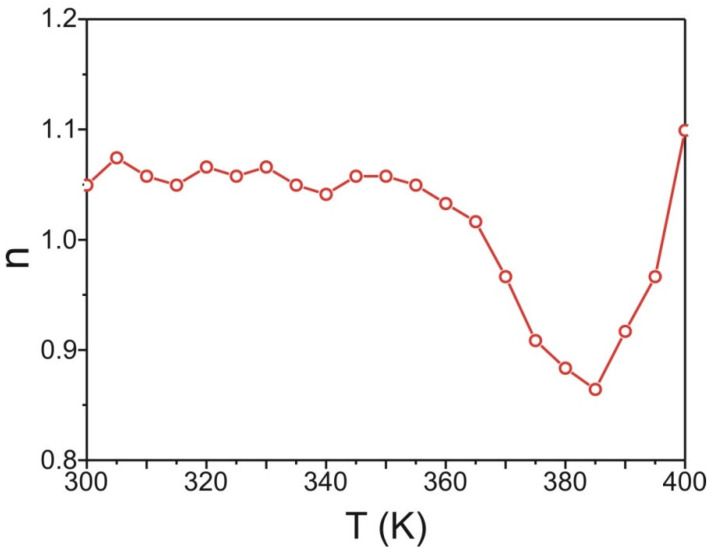
The temperature dependence of the exponent n for studied material.

The amorphous alloys correspond well with the 3D Heisenberg theory and critical exponents given by this approach are β = 0.365, γ = 1.386 and δ = 4.797. Based on these values and Equation (6), the n exponent at the Curie point should be 0.88, as it is shown in [18]. The n vs. T curve constructed for studied samples are shown in Figure 7. The analysis of this curve revealed that the value of exponent n for the specimen in a ferromagnetic state (below T_C_) is a bit higher than 1, while in a paramagnetic state (above T_C_), the n does not reach 2. It could be related to the presence of some crystalline phase traces in the material.

The value of exponent n at the Curie temperature is 0.86 and it is close to the theoretical value 0.88. The analysis provided above proved an existence of the second order phase transition in the Fe_60_Co_12_Gd_4_Mo_3_B_21_ alloy. It is well known that second order phase transition is expressed by the critical exponents. The evolution of such magnitudes as spontaneous magnetization M_S_, inverse magnetic susceptibility 1/χ and isothermal magnetization at the Curie temperature are strongly depended on critical exponents β, γ and δ, respectively. The relations between these magnitudes and critical exponents are given by following mathematical formula [19]:

(7) $$M_{S}(T)=M_{0}{\left(-\varepsilon \right)}^{\beta },\;\varepsilon <0,\;T<T_{C}$$

(8) $$\chi_{0}{\left(T\right)}^{-1}=\left(\frac{H_{0}}{M_{0}}\right)\varepsilon^{\gamma },\;\varepsilon >0,\;T>T_{C}$$

(9) $$M=DH^{\frac{1}{\delta }},\;\varepsilon =0,\;T=T_{C}$$

where ε = (T − T_C_)/T_C_ is reduced temperature, M_0_, H_0_ and D are critical amplitudes; H and M mean applied field and magnetization, respectively.

In order to determine δ based on calculated β and γ, Widom proposed a formula to obtain this exponent, today, well known as Widom scaling relation [20]:

(10) $$\delta =1+\frac{\gamma }{\beta }$$

The analysis of the Kouvel–Fisher plot revealed values of critical exponent β and γ, which equaled 0.361 ± 0.11 and 1.263 ± 0.11, respectively. Linear fitting of the ln(M) vs. ln(H) data allowed to reveal exponent δ = 4.52 ± 0.13, which is relatively close to it delivered by Widom scaling relation δ_W_ = 4.499. The calculated values of the critical exponents are close to those delivered by 3D Heisenberg theory. It means that short-range interactions dominate in produced alloy, which was previously studied in following papers [12,21]. The long-range quenched disorder observed in amorphous materials generates some smear of the ferromagnetic-paramagnetic transition. It was confirmed by M vs. T and the MCE studies, where there is a clearly visible large temperature range of transition. Taking into account mean-field model, the transition is observed at specific temperature. The revealed critical exponents allowed calculation of a theoretical n_t_ value of the exponent using relation (6) and their equaled 0.875. Calculated value is almost the same as this, revealed in Figure 6. Moreover, the Curie point was refined to 386.8 ± 0.1 K and corresponds excellently with previous studies based on M vs. T curve. Such high Curie temperature qualifies produced material as an active element in a heat pump.

## 4. Conclusions

In the present studies, we investigated amorphous ribbons of the Fe_60_Co_12_Gd_4_Mo_3_B_21_ alloy. The amorphous structure was confirmed by the X-ray diffraction and the Mössbauer spectroscopy. An investigation of magnetic entropy change revealed its value was 0.76 J (kg K)^−1^ under the change of external magnetic field 1T. The critical behavior studies in the vicinity of T_C_ resulted in the precise value of the Curie temperature for the studied sample and its critical exponents using the Kouvel–Fisher method. The calculated critical exponents corresponded well with Heisenberg theory and an occurrence of long-range disorder in sample was confirmed. The analysis of power field dependence of the magnetic entropy change revealed the n exponent. A relatively good agreement between the theoretical and experimental value of the exponent n determined on calculated critical exponents and experimental data, respectively, was detected.

## Figures and Tables

**Table 1:** The average value of the quadrupole splitting distribution (QS_1_), isomer shift of the single line (IS_1_) and its relative intensity (I_C1_), the relative intensity of the second and fifths line in sextet (A_2;5_), average hyperfine field <B_hf_>, average hyperfine field of components <B_hf_>_i_ their standard deviations Δ<B_hf_>_i_, parameters of linear dependence between (B_hf_)_i_ and IS_i_ (IS_0i_ and a_i_) and their relative intensities (I_i_) (i = 1, 2, C1) for each components, respectively, of Fe_60_Co_12_Gd_4_Mo_3_B_21_. alloys in the as-quenched state. Uncertainties for the last significant figure are given in brackets.

	Fe_60_Co_12_Gd_4_Mo_3_B_21_
	**Amorphous**
I_1_	0.95
(A_2;5_)_1_	3.0(1)
<B_hf_>_1_ [T]	9.66(7)
Δ(B_hf_)_1_ [T]	5.30(5)
(IS_0_)_1_ [mm/s]	−0.036(7)
a_1_ [mm/s^−1^T^−1^]	0.0019(6)
<B_hf_>_2_ [T]	15.4(5)
Δ(B_hf_)_2_ [T]	3.1(5)
(IS_0_)_2_ [mm/s]	0.32(8)
a_2_ [mm/s^−1^T^−1^]	−0.016(9)
<B_hf_> [T]	9.97(3)
	**Crystalline**
I_C1_	0.01
QS_1_ [mm/s]	0.07(2)
IS_1_ [mm/s]	0.017(7)

## Data Availability

The data presented in this study are available on request from the corresponding author.
